# Spark Plasma Sintering Apparatus Used for High-temperature Compressive Creep Tests

**DOI:** 10.3390/ma13020396

**Published:** 2020-01-15

**Authors:** Barak Ratzker, Sergey Kalabukhov, Nachum Frage

**Affiliations:** Department of Materials Engineering, Ben-Gurion University of the Negev, P.O.B. 653, Beer-Sheva 84105, Israel; ratzkerb@post.bgu.ac.il (B.R.); kalabukh@bgu.ac.il (S.K.)

**Keywords:** spark plasma sintering apparatus, compressive creep test, stress exponent, electric current

## Abstract

Creep is a time dependent, temperature-sensitive mechanical response of a material in the form of continuous deformation under constant load or stress. To study the creep properties of a given material, the load/stress and temperature must be controlled while measuring strain over time. The present study describes how a spark plasma sintering (SPS) apparatus can be used as a precise tool for measuring compressive creep of materials. Several examples for using the SPS apparatus for high-temperature compressive creep studies of metals and ceramics under a constant load are discussed. Experimental results are in a good agreement with data reported in literature, which verifies that the SPS apparatus can serve as a tool for measuring compressive creep strain of materials.

## 1. Introduction

**Figure 1:**
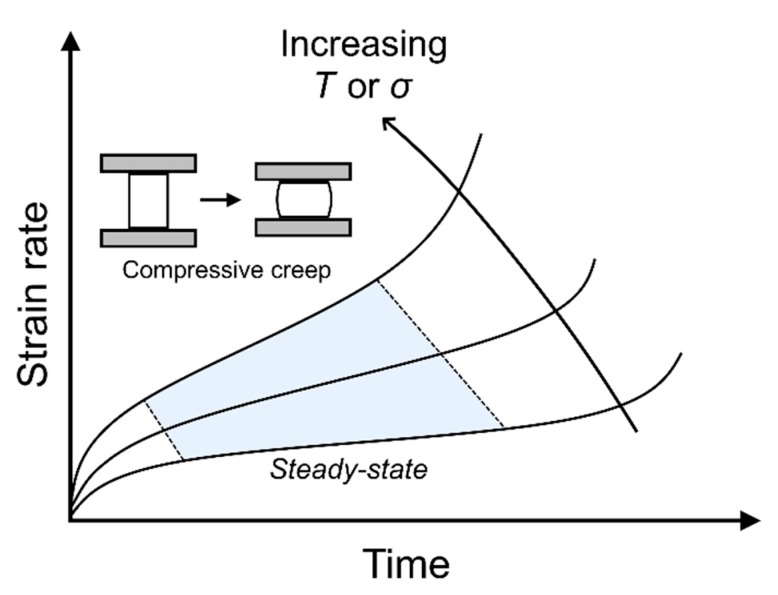
Schematic illustration of creep curves; strain rate increases with temperature or applied stress.

Creep is the continuous deformation of a material subjected to a constant load or stress, often lower than its yield strength. It is a process that is very sensitive to temperature, generally considered as high-temperature deformation, which occurs at roughly ≥ 0.5 *T/T_m_* (where *T/T_m_* in K is the homologous temperature) [1]. It is an imperative issue, which could lead to failure of engineering materials that are under stress and exposed to high-temperature environments. Compressive creep tests are a convenient method for investigating high-temperature deformation, especially for strong and brittle refractory materials such as ceramics [2,3,4]. Creep generally includes three different stages: the primary, secondary and tertiary stage (Figure 1). The primary and tertiary are the initial and final stages, in which there is a transient strain rate that decelerates to a steady rate or accelerates up to failure by rupture, respectively. Meanwhile, during the second stage, termed steady-state creep the strain rate is constant (or nearly constant). The creep rate in this stage is used to evaluate high-temperature deformation and creep behavior of materials.

Measuring steady-state creep rate is important for prediction of the service lifetime for structural components, as well as understanding the micromechanics and metallurgical aspects of high-temperature deformation. Accurate strain rate measurements make it possible to determine creep parameters and identify the operating deformation mechanisms [5,6]. Creep rate generally depends on either external conditions, such as temperature and applied stress, or material properties, such as grain size and presence of precipitates or dopants.

Creep strain rate is most sensitive to the temperature and applied stress and can be described by an Arrhenius exponential and power-law dependency, respectively. The basic power-law creep equation is generally presented as:

(1) $$\dot{\varepsilon }=A\sigma^{n}\exp\left(-\frac{Q}{RT}\right)$$

where *A* is a constant, σ is the applied stress, *n* is the stress exponent, *Q* is the apparent activation energy, *R* is the gas constant and *T* is the temperature. The experimental creep rates can then be analyzed according to their dependency on stress and temperature which allows to determine values of *Q* and *n*.

(2) $$\ln\dot{\varepsilon }=\ln A+n\ln\sigma -\frac{Q}{RT}$$

The creep apparent activation energy (*Q*), which is inherent to the material, reflects the diffusion processes taking place at elevated temperatures and determines the creep temperature dependence. Diffusion also acts as an accommodating process to grain boundary sliding and dislocation creep mechanisms at lower temperatures [6,7]. The stress exponent (*n*) reflects the sensitivity to the applied stress and creep dependence on the load. According to Equation (2), *Q* and *n* can be determined by either measuring creep at different temperatures under same applied load, or at the same temperature under different applied loads, respectively. A proper creep test setup must allow precise gauging of the temperature and stress applied to the sample with accurate measurements of the axial displacement [8].

Spark plasma sintering (SPS) is an advanced pressure-assisted sintering technique, which utilizes an electric current for heat generation within conductive tooling and powder compacts [9,10]. This combination makes it possible to achieve excellent sintering capabilities of many metallic, ceramic and composite materials [11]. To track the densification progress, the SPS apparatus is equipped with a strain gauge and built in LVDT. It records the punch displacement every second with an accuracy of ~1 µm (depending on the SPS system). Consequently, the SPS apparatus encompasses all the necessary components (including temperature and load control) to perform compressive creep tests. Therefore, it was suggested that the SPS apparatus could be used as an accurate creep testing tool [12]. Such capabilities have already been demonstrated for both ceramics [12,13] and metals [14,15]. It is worthy to note that SPS creep test results have already shown good agreement with previously reported data obtained by conventional testing methods for the same materials at similar temperature/pressure ranges [12,13,14,15]. Moreover, it has been proposed that the SPS apparatus’ ability to apply an electric current to the sample makes it possible to investigate to some extent electro-plastic effects in conductive materials during high temperature deformation [14,15]. This is particularly important for SPS, because it could also be directly connected to the enhanced sintering behavior of conducting materials [16,17].

In the present study, we describe in detail the use of an SPS apparatus for creep investigation of metals and ceramics, highlight the advantages and disadvantages of this method, and provide prominent experimental examples of creep tests performed by an SPS apparatus applied as a creep testing device.

## 2. Test Setup and Procedure

### 2.1. SPS Apparatus Technical Details

The SPS apparatus requires practically no modification to serve as a creep testing device in compliance with ASTM technical standards [18]. The following description is based on an FCT system SPS (FCT Systeme GmbH, Rauenstein, Germany), but would generally be the same for other SPS machines from other manufacturers. The system allows to easily set the testing parameters (i.e., load, temperature) and track them continuously in 1 s intervals, along with many other parameters derived from them (e.g., punch displacement, current, voltage). The specifications of the SPS apparatus used in this study are given in Table 1.

To perform a creep test, a columnar sample is set at the center between the punches. To ensure that the sample is placed in an unconstrained manner, the initial and final sample dimensions should be considered prior to the test. The relative punch displacement (RPD) is monitored with an accuracy of ~1 µm for an HP-D10 FCT System (this may vary for other machines). The measured RPD can be converted to strain, simply by dividing it by the initial sample height while taking in account the thermal expansion of the material. The corresponding creep rate can then be determined from the slope or derivative of the strain curve [12,14].

### 2.2. Test Configurations and Temperature Considerations

**Figure 2:**
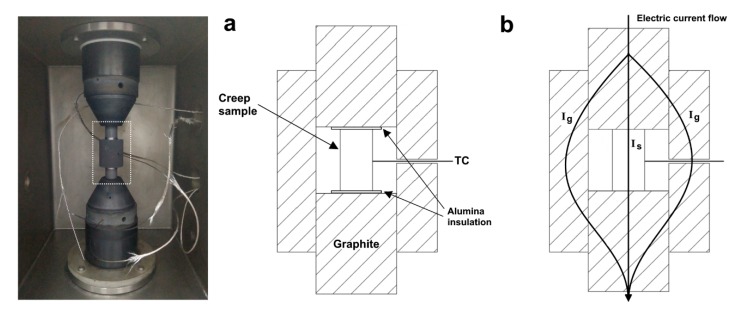
Schematics and images of configurations creep testing (**a**) without and (**b**) with electric current applied to the sample. The electric current flow is portrayed schematically.

The temperature in the SPS apparatus is typically measured using the built-in system pyrometer or thermocouples. For the best accuracy during creep tests, it was suggested to place a thermocouple (C, K or S type for our system) in direct contact with the sample surface (see Figure 2). Temperature distribution in SPS is a known issue which also depends on the tooling configuration [19,20]. If there is only resistive heating of the sample during creep tests of conducting materials (i.e., tooling without a surrounding die as was used in our previous studies [14,15]) the temperature deviations may be relatively large due to significant heat loss from the sample surface. To mitigate this, it is suggested in the present study to apply an electric current while using the graphite die, like the configuration discussed in [21]. The minor disadvantage in this case is that the electric current value applied by the SPS apparatus splits between the die and sample. The actual current applied to the sample (I_s_) can then only be estimated according to the relative electrical resistance of the tooling and the sample according to the Kirchhoff’s law. Thus, the different heating configurations for SPS apparatus creep tests are as depicted in Figure 2. In which conductive materials can be resistively heated by passage of an electric current as well as by heat convection and radiation from the graphite punches and die, respectively. The electric current can be avoided by separating the sample from the graphite by the means of a ceramic insulator, such as alumina. When tests are conducted at relatively high temperatures a graphite felt should be placed around the die to further mitigate heat dissipation.

**Figure 3:**
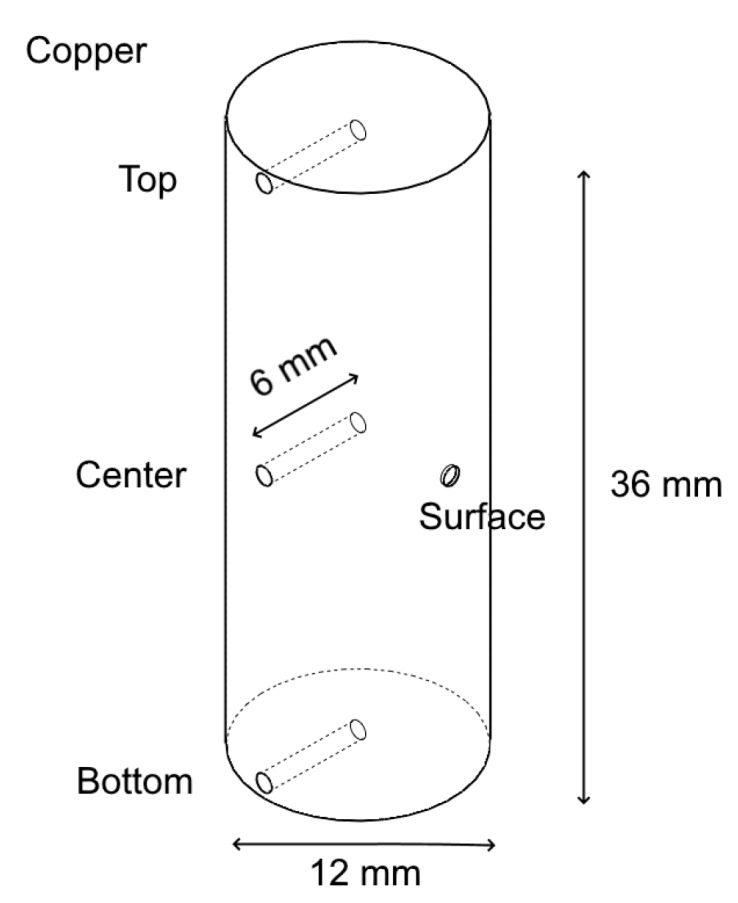
Schematic illustration of copper sample and the holes used for temperature measurements.

To estimate possible temperature distributions in samples tested with different tooling configuration, a special set of temperature measurements was conducted. The measurements were performed on a cylindrical copper sample with several 1.5 mm holes drilled 6 mm deep into the center, top and bottom (Figure 3). Using two thermocouples simultaneously, multiple temperature measurements (in the 450–550 °C temperature range) were performed. Each measurement was taken when the temperature was stabilized. The results for both tooling configurations are summarized in Table 2. The temperature was defined according to the thermocouple located on the sample surface. When testing the difference between the center and top/bottom regions the temperature was defined according to the thermocouple located in the center of the sample.

The tests revealed the presence of a temperature gradient within the sample. In the case of a sample insulated from the electric current (Figure 2a), the radial temperature difference, between the surface and center is about ~15 °C. This difference in temperature would exists in any conventional creep test apparatus, since the sample is heated from the outside. As for the case with the electric current, the measurements showed a larger difference of roughly ~20–30 °C between the center and surface. However, we believe that these values should be taken with a grain of salt. The temperature measured in the center may be inaccurate due to higher current density developing around the hole. Even a small addition of current would cause significant extra localized heating around the thermocouple, making these measurements erroneous. In fact, considering the rate of heat loss from the surface, we expect that the real temperature gradient is roughly the same as was observed for the insulated sample (or even lower since the whole sample is heated by the current), but in the reverse direction. Additionally, there is also a difference between the top and the bottom of the sample This difference can be attributed to the SPS apparatus design in which the upper punch is the positive electrode and usually hotter. As shown by Sweidan et al. [21], approaches can be taken to minimize temperature deviations and achieve more accurate testing.

The uncertainty of the temperature measurements may be problematic to directly compare results of creep tests with and without an electric current and to discuss the effect of electro-plasticity. Furthermore, it should be considered that for such creep tests at elevated temperatures joule heating effects also contribute to deformation and stress relaxation and cannot be easily de-coupled from the electro-plastic effect [22]. This issue will be further considered in the next section.

### 2.3. Creep Testing Procedure

**Figure 4:**
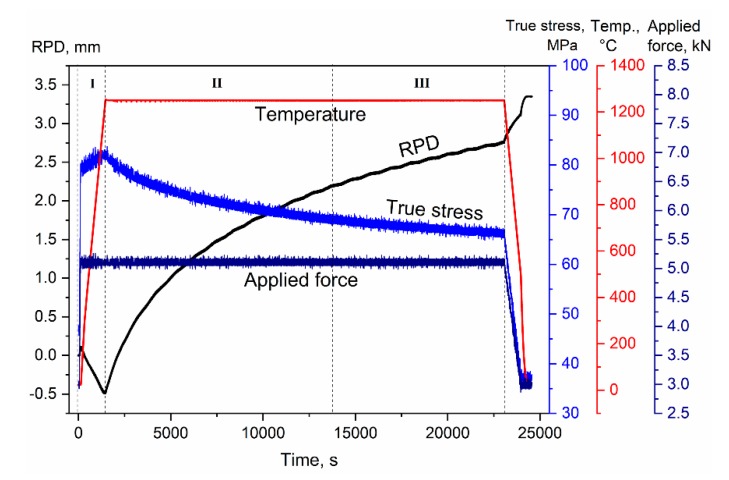
Creep test variables recorded by the SPS system (true stress is calculated). The different test stages (segments **I**, **II** and **III**) related to conducting a creep test are depicted [12]. Reproduced with permission of Elsevier.

The creep test itself is performed in a relatively simple manner, by setting the designated temperature and load (pressure is calculated according to sample cross-section) and tracking RPD. The test can be conducted under constant temperature or load, but also with various pressure or temperature steps [15], to obtain multiple creep rate measurements from a single sample. An example for a testing procedure of alumina (at 1250 °C under 80 MPa), including all relevant experimental data necessary for creep evaluation, is presented in Figure 4. The heating stage **I** (Figure 4), is conducted prior to the creep test, while a minimal or designated test force is applied. At this stage, the negative RPD indicates the thermal expansion of the graphite tooling system and stainless-steel pistons. In segments **II–III** (Figure 4), the recorded displacement reflects only sample deformation after a mechanical and thermal equilibrium have been reached. In segment **II** there is a rapid decrease in the strain rate, while in segment **III** the strain rate is practically constant. Thus, segments **II–III** are considered primary and steady-state creep, respectively. It should be noted that the steady-state mentioned above (segment **III**) is sometimes a quasi-steady-state, due to the continuous reduction of true stress during creep (Figure 4) as well as concurrent grain growth [23,24]. This would be more of an issue at high strain rates under testing conditions of relatively high temperatures or applied stress [12].

**Figure 5:**
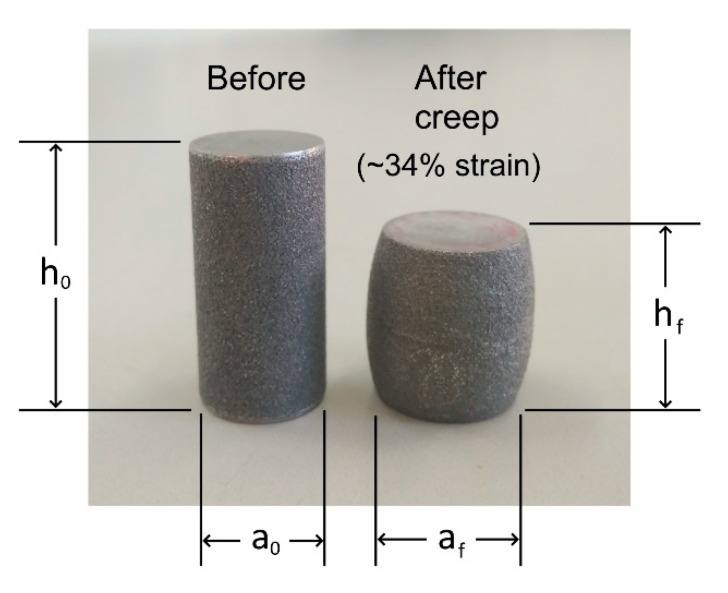
Example of AlSi_10_Mg samples before and after creep test (~34% strain) at 225 °C under an initial applied pressure of 130 MPa.

Nevertheless, using an SPS apparatus for creep tests has several technical limitations. A minimal load of 3 kN must be applied during the test in order to receive displacement recording. This limits the sample size and determines the minimal applied stress. Furthermore, the SPS system cannot be set for a certain stress or constant strain rate and thus the accurate measurement of high-temperature compressive strength cannot be performed. Since the SPS apparatus typically only allows to apply a constant load, the actual stress on the sample continuously decreases during the test as the sample cross-section expands with increasing strain (Figure 5). This issue is treated by calculating the true strain (for any given moment) [25].

(3) $$\varepsilon_{t}(t)=\ln\frac{l_{t}}{l_{0}}$$

where $\varepsilon_{t}$ is the true strain, $l_{t}$ is the current specimen height at a given point in time and *l*_0_ is the initial specimen height. Considering volume conservation (before excessive cavitation at final stages of creep), the true stress *σ_t_* can then be calculated.

(4) $$\sigma_{t}=\frac{F}{a_{0}^{2}}\left[\exp(\varepsilon_{t})\right]$$

where *F* is the applied load and $a_{0}^{2}$ is the initial sample cross-section area.

Thus, the strain rate during compressive creep tests can be summarized as the following equation.

(5) $$\dot{\varepsilon }=A{\left(\frac{F}{a_{0}^{2}}\left[\exp(\varepsilon_{t})\right]\right)}^{n}\exp\left(\frac{-Q}{RT}\right)$$

## 3. Results and Discussion

### 3.1. Creep Testing of Metals

As discussed in the previous section, metals (and other conductive materials) can be tested by heating generated from the graphite tooling while insulated from the punches or including resistive heating within the sample by allowing passage of the electric current through the sample. In our previous study on copper [14], it was shown that SPS-measured creep rates without the current agree quite well with results of conventional tensile creep tests that have been performed on copper under at the same temperature range (400–600 °C). The slope against the reciprocal of temperature is similar, which means that the creep apparent activation energy *Q* is the same. In this case, *Q* was equal to about 110 kJ/mol, which corresponds to vacancy migration (dislocation motion in vacancy saturation) [26]. This validated the SPS apparatus as an accurate creep testing tool. Nevertheless, for the case with applied electric current we used only resistive heating without surrounding die and the obtained results regarding electro-plastic effect may be questionable.

**Figure 6:**
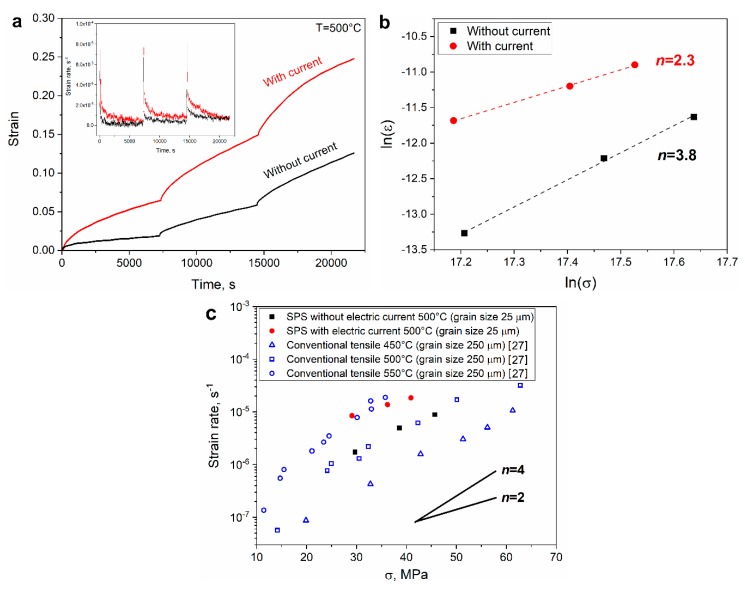
(**a**) Example of copper creep test at 500 °C with three load steps with and without an applied current, the corresponding strain rate in the insert; (**b**) the creep rates as a function of stress (natural log) with the slope value *n* indicated and (**c**) as a function of stress compared with other reported results [27] for tensile creep of copper at various temperatures (notice the different grain size).

Therefore, in the present study, we performed additional creep experiments on similar copper samples with a surrounding die, which allows to lower the temperature gradient. These isothermal creep tests were conducted with load increments of 30, 40 and 50 N, while maintaining a constant temperature of 500 °C. This makes it possible to investigate the stress dependence and determine the stress exponent *n*. Each of these tests was performed in both possible configurations, with and without an electric current. The obtained creep curves are presented in Figure 6a, and the calculated creep rates are presented as a function of stress in Figure 6b. It was found that the value of *n* without the current was close to 4 which agrees with the known values for stress exponent of copper at relatively low stress (<100 MPa) and intermediate homologues temperatures [27]. While the value with the applied current was significantly lower, at around 2.3 (which is very low for copper). It has to be pointed out that stress exponent does not depend a relatively small temperature difference. See for instance a comparison with reported data for copper creep (tensile) at various temperatures (Figure 6c). Thus, the considerable difference of in the stress exponent could only be attributed to some electro-plastic effect which may affect the creep mechanism. Nevertheless, it is quite difficult to de-couple between the additional heating and electro-plastic effect, but the latter clearly has a contribution to susceptibility to creep [28,29]. Such results help to explain enhanced densification during SPS of conducting materials under influence of an electric current [16]. It perhaps may be possible to gain a deeper understanding on the matter by performing creep tests with different electric currents by altering the SPS tooling [30].

### 3.2. Creep Testing of Ceramics

**Figure 7:**
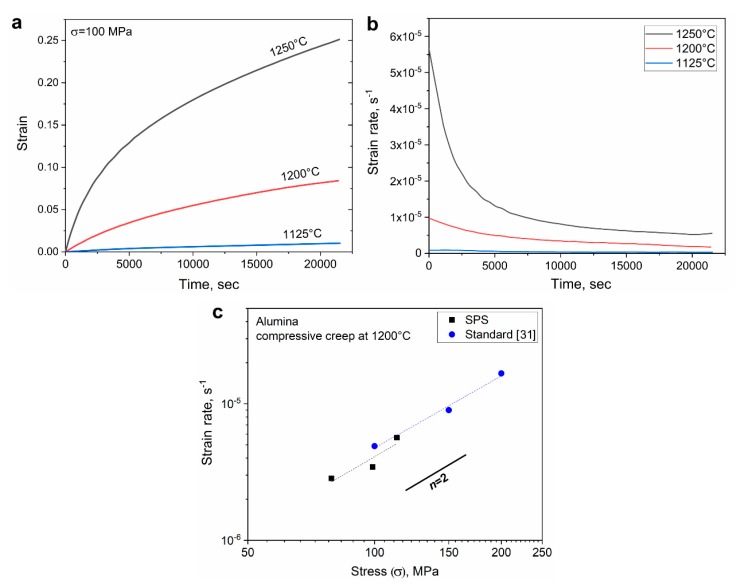
(**a**) Example of alumina creep tests strain under an applied stress of 100 MPa at the 1125–1250 °C temperature range and (**b**) corresponding strain rates; (**c**) comparison of creep rates (at 1200 °C and varying stress) measured for alumina by an SPS apparatus in compression (black squares) and conventional compression testing equipment [31] (blue circles).

In the case of insulating ceramics, a configuration involving the graphite die must be used (Figure 2). However, it should be taken into account that some ceramics are still conductive, such as ZrN, and would involve an effect of electric current within the sample [17]. Also, under strong electric fields there may also be field effects in ceramics that can influence the creep behavior [22]. SPS was applied for a creep study of polycrystalline MgAl_2_O_4_ (for the first time) to clarify the deformation mechanisms at high temperature under relatively high stress [13]. For instance, it was found that the apparent activation energy decreases with increased applied stress. To further validate the creep measurements obtained by an SPS apparatus, it was used to examine alumina, perhaps the most widely researched ceramic material. Alumina single stage creep curves from a previous study [12], performed at various temperatures (in the range of 1125–1250 °C) and under an applied stress of 100 MPa, are presented in Figure 7a. Corresponding strain rates (according to the derivative over time) are presented in Figure 7b. The dramatic effect of the temperature on the total strain and strain rate (slope) can be easily observed. Furthermore, alumina creep rate values obtained at 1200 °C under varying loads are presented as a function of stress, alongside values from a study conducted in compression by Bernard-granger et al. [31] (Figure 7c). Both studies were performed on alumina with a similar fine grain size (0.5 and 0.42 µm, respectively). Here, as well, there is a good agreement between creep measurements by SPS and data reported in literature. The slope reflects the sensitivity to the applied stress and corresponds to a stress exponent *n* of about 1.8 which is close to 2 and established for fine-grained alumina [12,32].

### 3.3. High-Pressure Creep Tests

**Figure 8:**
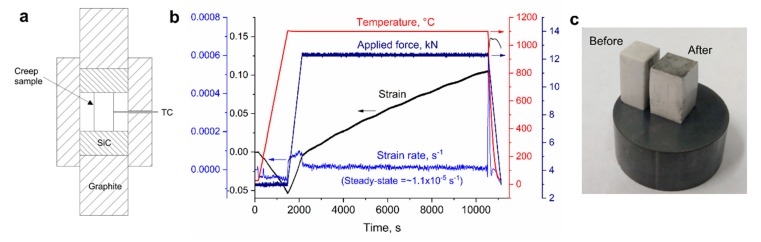
(**a**) Schematic illustration of hybrid SiC-graphite tooling for high-pressure experiments; (**b**) an example for a high-pressure creep test of alumina (sample size 5 × 5 × 10 mm, initial applied stress 400 MPa); (**c**) appearance of samples before and after the high-stress creep test.

In some cases, such as for Ni-based superalloys, there is a lot of interest in creep properties under high-stress conditions [33,34,35]. Typically, it is difficult to examine high-temperature deformation under high applied stress. However, one of the advantages of the SPS apparatus as a creep testing device is that it can allow tests under stresses of hundreds of MPa, using proper high-pressure tooling such as SiC punches [12]. The materials that can be investigated will depend on properties of the high-pressure tooling. For instance, SiC has significantly higher resistance to creep [36] compared to oxide ceramics and can be applied for such tests under high pressure. This was demonstrated for alumina, which was tested under an applied pressure of 400 MPa (sample dimensions 5 × 5 × 10 mm), as presented in Figure 8. These types of tests can clarify creep mechanisms and unique mechanical behavior of materials subjected to a combination of high temperature and stress.

## 4. Conclusions

An SPS apparatus can be used for studying high temperature mechanical properties, particularly compressive creep of metals and ceramics. In this apparatus, a wide range of temperatures and pressures can be applied, and all the necessary data for creep tests can be easily acquired. However, the SPS apparatus, as a tool for mechanical testing, has some technical limitations, including the mandatory minimal load of 3 kN, the lack of a possibility for tensile testing, and the fact that only a constant load regime can be applied. Nevertheless, different tooling configurations may be used so that conductive materials can be tested with or without an applied electric current. This affects the different temperature gradients which exist in the sample, especially when an electric current is applied. Nevertheless, the current that the SPS apparatus utilizes can make it possible to investigate electro-plastic effects to some extent. Furthermore, creep tests under relatively high applied stress (in the range of few hundreds MPa) can also be realized by the SPS apparatus. Several creep test results obtained by an SPS apparatus were presented, and experimental creep results for metals (copper) and ceramics (alumina) proved the accuracy of this device for creep testing of engineering materials. Thus, the SPS apparatus can serve as a relatively simple and convenient method for a wide range of creep testing of both metals and ceramics.

## Figures and Tables

**Table 1:** Lab-scale HP-D 10 FCT system SPS apparatus specifications relevant to creep testing.

Temperature Range, °C	Pressing Force, kN	Displacement Resolution, mm	Programmable Test Segments	Electric Current Applied to Sample	Chamber Atmosphere
Up to 2400	3–100 (stress depends on sample cross-section)	0.001	Yes	Possible for conductive samples	Vacuum (10^−2^ mbar) with argon flow

**Table 2:** Temperatures measured at various regions of copper sample for the different testing configurations with or without passage of an electric current.

Test Configuration	Temperature at the Surface, °C	Temperature at the Center, °C	Temperature at the Top, °C	Temperature at the Bottom, °C
With electric current	450	473 *		
	500	524 *	515 *	501 *
	550	579 *		
Insulated from the electric current	450	438		
	500	486		
	550	536		

* Suspected to be inaccurate due to high current density causing a temperature rise around the thermocouple locations.
